# Malt Extract

**Published:** 1870-12

**Authors:** Albert E. Ebert


### MALT EXTRACT.

By ALBERT E. EBERT.

The present time in the history of Pharmacy, may rightly be styled the era of "scientific specialities." The latest efforts in this direction are malt extracts. Two classes of preparations under this title, are met with in the market, having widely different properties: one variety may be classed among alcoholic beverages, the other is purely saccharine in nature. To the first belong the preparations of Hoff and of Koch, the latter bearing the name of Liebig. Hoff's extract has obtained a ready sale by aid of extensive advertising, a fact which is surprising, after the exposures made by Hager and Wittstein. These chemists determined it to be simply a good article of Brewer's beer, having an alcoholic strength of about three per cent., with an addition of marsh mallow root, coriander, staranise, and grains of paradise, sweetened with glycerine or sugar, flavored with the oils of lemon and orange, and colored with burnt sugar. Koch's preparation purports to be similar to Hoff's, with the prominent advantage well set forth in the words of the proprietor, as follows: "Owing to the facilities we have in manufacturing in this country, we are enabled to sell Koch's extract at the astonishing low rate of $3.00 per dozen, or thirty cents for single bottle." He magnanimously offers to suffering humanity, lager beer at only six times its retail value, while Hoff charges about fifty per cent. more for the same article! How thankful we should be for his fortunate "advantage," and we must also feel deeply grateful after perusing the circular which accompanies this great medicine. "The most eminent medical authorities in Europe, as well as in this country, agree that this new tonic is the best *dietetic and healing remedy* known to modern science, combining both the merits of a nutritious and palatable beverage, and the virtues of an unfailing medicine for general diseases of the lungs, the chest, and the throat, while to those in good health it serves as a *pleasant table drink*, promoting their digestion, and restoring and invigorating their appetite—(for what?) This malt extract offers the most beneficial relief to the sick in all cases where the stomach, the lungs, or the throat are affected."

The reader will say, "but medical men do not recommend such nostrums." In reply, we will say that we have had prescriptions for this beer from men who lay claim to a scientific training, and have, in each case, advised the patient or messenger to procure the same by the gallon at some saloon, where they may obtain it at first cost

This proves the truth of the saying, "there is something in a name," and Malt Extract is equally as good a term as the celebrated "Stomach Bitters," whose chief advantage is the quantity of alcohol hidden by the high-sounding titles, and made into a fancy tippling drink by the aid of a few bitter roots, herbs and spices. But the greatest shame of all is the frequent recommendations and endorsement by letters, which are published by the factors of these vile nostrums, and scattered broadcast to the public, and these testimonials are frequently from men who occupy prominent positions in science—yes, even teachers in medical colleges. But, alas! we were lately astounded by seeing such an endorsement from a gentleman occupying the chair of Pharmacy, in the St. Louis College of Pharmacy. His private endorsement might have been overlooked, but when he attached to his name his position in the College, to give it extra weight, he gave an insult to the entire organization in which he holds the office of a teacher; the remedy of making a few examples by excommunication would go far towards stopping this prostitution of our profession to such base uses.

Of the second class of malt extracts, sometimes called Liebig's, we have seen two samples, from German manufacturers, Ed. Loeflund, and Dr. H. E. Linck, both of Stuttgard. They were put up in patent medicine style, each claiming originality in the process of manufacture. This point is questionable, as malt extract has been officinal in the London, Edinburgh, and Belgium Pharmacopœias, and in long use in Germany under the name of malt sugar (Gerstenzucker.)

Prof. Liebig does not lay any claim to the discovery or introduction of this preparation; we have heard him, during his lectures denounce this attachment of his name to those extracts, it having been done in opposition to his wishes by parties who hoped to increase their sales by this seeming endorsement of their articles. We have lately made the malt extract, at the urgent request of physicians, and give herewith the process, so that pharmacists may prepare it themselves, instead of relying upon the specialist to supply it at exorbitant prices:

Take of barley malt, kiln dried, 10 lbs., av.
Water, q. s.

The malt may be obtained at the malt-houses or breweries, by the bushel; reduce it by means of the drug-mill, so that it will pass through a No. 20 sieve, and add to the meal a sufficient quantity of cold water to form with it a soft dough; then add two gallons of hot water, and apply heat so as to raise the temperature of the mixture to 150 degrees, or not to exceed 158. Maintain this temperature, with occasional stirring, for several hours, or until the whole of the starch is converted (by means of the diastase of the malt) into dextrine and glucose. The absence of starch can be ascertained by the application of tr. iodine to a small quantity of the liquor, when, if the starch has been wholly converted, no blue coloration will be evident. Then express the liquor rapidly, and pass it through a strainer. This is the most difficult part of the process, as it speedily clogs the strainer; this can be averted to some extent, by making a pulp by means of water, from common unsized paper, or filtering paper, and mixing this pulp with the expressed liquid, previous to straining. The perfectly clear fluid is finally to be evaporated by means of a water bath, to the consistence of a thick syrup, having the sp. gr. 1,500, or approximately one pint, weighing 1½ pounds, average.

This extract has an agreeable, syrupy taste, and contains, besides the sugar of the malt, dextrine, albumen, and the phosphates of the grain. In very hot summer weather, it is liable to go into fermentation, but this can be prevented by the addition of a small quantity of glycerine.—*The Pharmacist.*

